# Dissecting components of the *Campylobacter jejuni fetMP-fetABCDEF* gene cluster under iron limitation

**DOI:** 10.1128/spectrum.03148-23

**Published:** 2023-12-14

**Authors:** Tomas Richardson-Sanchez, Anson C. K. Chan, Brendil Sabatino, Helen Lin, Erin C. Gaynor, Michael E. P. Murphy

**Affiliations:** 1 Department of Microbiology and Immunology, The University of British Columbia, Vancouver, British Columbia, Canada; Universidad Andres Bello, Santiago, Chile

**Keywords:** *Campylobacter jejuni*, iron transport, Fet system genes, thiol-disulfide oxidoreductase, X-ray crystallography

## Abstract

*Campylobacter jejuni* is a leading cause of bacterial gastroenteritis worldwide. Acute infection can be an antecedent to highly debilitating long-term sequelae. The expression of iron acquisition systems is vital for *C. jejuni* to survive the low iron availability within the human gut. The *C. jejuni fetMP-fetABCDEF* gene cluster is known to be upregulated during human infection and under iron limitation. While FetM and FetP have been functionally linked to iron transport in prior work, here we assess the contribution of each of the downstream genes (*fetABCDEF*) to *C. jejuni* growth during both iron-depleted and iron-replete conditions. Significant growth impairment was observed upon disruption of *fetA*, *fetB, fetC*, and *fetD*, suggesting a role in FetMP-mediated iron acquisition for each encoded protein. FetA expression was not dependent on the presence of FetB, FetC, FetD, FetE, or FetF. The functions of the putative thioredoxins FetE and FetF were redundant under iron-limited growth, requiring a double deletion (Δ*fetEF*) to exhibit a growth defect. *C. jejuni* FetE was expressed, and the structure was solved to 1.50 Å, revealing structural similarity to thiol-disulfide oxidases. Functional characterization in biochemical assays showed that FetE reduced insulin at a slower rate than *Escherichia coli* Trx and that together, FetEF promoted substrate oxidation in cell extracts, suggesting that FetE (and presumably FetF) are oxidoreductases that can mediate oxidation *in vivo*. This study advances our understanding of the contributions of the *fetMP-fetABCDEF* gene cluster to virulence at a genetic and functional level, providing foundational knowledge toward mitigating *C. jejuni*-related morbidity and mortality.

**IMPORTANCE:**

*Campylobacter jejuni* is a bacterium that is prevalent in the ceca of farmed poultry such as chickens. Consumption of ill-prepared poultry is thus the most common route by which *C. jejuni* infects the human gut to cause a typically self-limiting but severe gastrointestinal illness that can be fatal to very young, old, or immunocompromised people. The lack of a vaccine and an increasing resistance to current antibiotics highlight a need to better understand the mechanisms that make *C. jejuni* a successful human pathogen. This study focused on the functional components of one such mechanism—a molecular system that helps *C. jejuni* thrive despite the restriction on growth-available iron by the human body, which typically defends against pathogens. In providing a deeper understanding of how this system functions, this study contributes toward the goal of reducing the enormous global socioeconomic burden caused by *C. jejuni*.

## INTRODUCTION

*Campylobacter jejuni* is a Gram-negative ε-proteobacterium and a leading cause of bacterial diarrheal disease worldwide. *C. jejuni* is a commensal in the intestinal mucosa of many wild and domesticated animals, commonly colonizing farmed poultry flocks and then undergoing zoonotic transmission to humans, by, for example, the consumption of undercooked poultry or cross-contamination of other food with raw poultry juice (1, 2). Acute human infection results in severe watery to bloody diarrhea and can also be an antecedent to highly debilitating long-term sequelae, such as inflammatory bowel diseases and autoimmune disorders (3 – 5). The high socioeconomic burden and impact on human health have been made worse by the inability to produce an effective vaccine and the increasing levels of antibiotic resistance in isolates from both hospitals (6, 7) and poultry meat (8).

Successful *C. jejuni* colonization of the human intestinal mucosa is dependent on a range of factors, including the expression of systems to acquire essential micronutrients such as iron. Recent transcriptional studies on *C. jejuni* have demonstrated the upregulation of an eight gene cluster (*CJJ81176_1649–1656,* hereinafter named *fetMP-fetABCDEF*), during human infection (9). Also, our group has shown increased expression of *fetMP-fetABCDE* upon exposure to human fecal metabolites (10), indicating a likely role in pathogenesis. The two upstream genes, *fetM* (*CJJ81176_1649*) and *fetP* (*CJJ81176_1650,* also known as *p19*), encode the FetMP iron transport system, which has been shown to be important for growth under iron-limited conditions (11, 12). The six downstream genes *fetABCDEF* (*CJJ81176_1651–1656*) have not previously been characterized individually, but collectively have been shown to be strongly upregulated alongside *fetMP* during iron restriction and upon deletion of *fur*, which encodes the ferric uptake regulator protein (13 – 15). Two other studies have observed *C. jejuni* growth defects under iron restriction upon disruption of the *fetMP-fetABCDEF* gene cluster, either by deletion of *fetP* (11) or, as our groups recently demonstrated, simultaneous deletion of all six downstream genes (Δ*fetABCDEF*) (10), with growth defects restored upon iron supplementation or complementation with the wild-type (WT) gene cluster. Additionally, *C. jejuni* exhibits a biphasic phenotype to the antibiotic streptomycin. Rather an unimodal concentration-dependent inhibitory effect, wild-type *C. jejuni* exhibits streptomycin tolerance at moderate antibiotic concentrations. This tolerance is lost upon the deletion of *fetABCDEF* but can be restored by supplementing the deletion strain with iron, supporting the role of this cluster in iron metabolism. *C. jejuni* Δ*fetABCDEF* also has increased acid sensitivity and higher resistance to oxidative stress (10). These transcriptional and phenotypic studies link *fetABCDEF* to a role in *C. jejuni* pathogenesis, providing greater impetus to investigate each individual component of the *fetMP-fetABCDEF* gene cluster.

**Fig 1:**
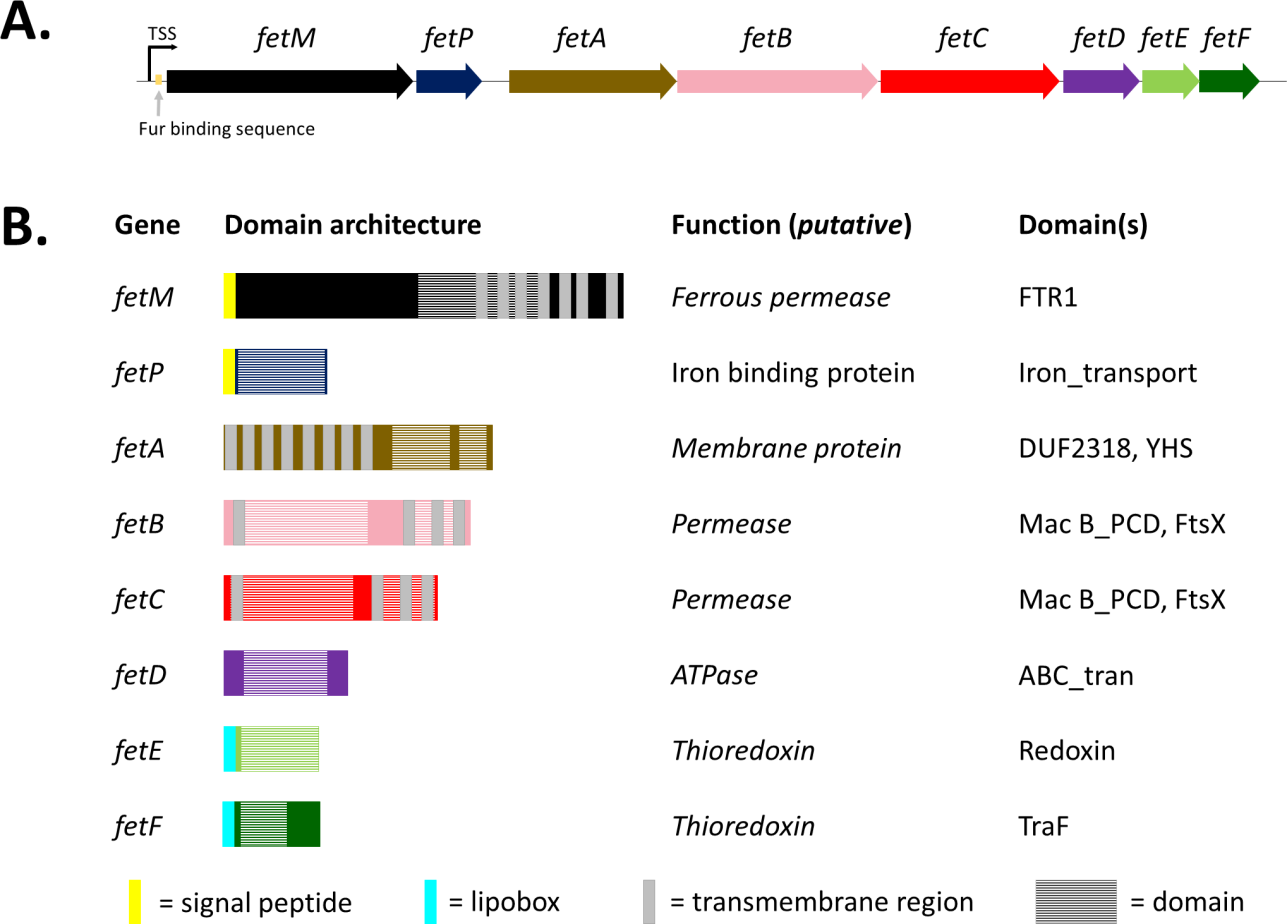
Putative operonic structure, domain architecture, and model with predicted functions of the *fetMP-fetABCDEF* gene cluster. (**A**) Proposed operon structure in *C. jejuni* wild-type strain 81–176 with primary transcription start site (TSS) and Fur binding sequence. (**B**) Gene function and domain architecture. Signal peptides and protein lipidation sites (lipoboxes) predicted by SignalP-5.0, transmembrane helices by TMHMM Server v2.0, and domain predictions by Pfam 32.0.

Gene clusters homologous to *fetMP-fetABCDEF* have been identified in 33 diverse bacterial species across six phyla, including 21 species that are associated with human disease (10). The *C. jejuni fetMP-fetABCDEF* gene cluster spans a genomic region of 8.1 kb and consists of two upstream genes (*fetMP*) separated from six downstream genes (*fetABCDEF*) by an 82 base intergenic region (Fig. 1A). Upstream of the *fetM* start codon is a Fur binding sequence (10 bases upstream) and a putative primary transcription start site (54 bases upstream) (16), suggesting *fetMP-fetABCDEF* may be transcribed as one operon.

*In silico* domain analysis of the proteins encoded by *fetMP-fetABCDEF* (Fig. 1B) allows the identification of putative functions in cases where functional studies on the proteins or their homologs are lacking. FetM is yet to be characterized in *C. jejuni*, although the *Escherichia coli* homolog has been demonstrated to be an iron transporter of the “oxidase-dependent iron transporter” family (17, 18). FetP has been characterized as a periplasmic iron binding protein in *C. jejuni* (11), as well as in *E. coli* (18), *Bordetella* (19), and *Yersinia pestis* (20). The FetMP iron uptake system was suggested to import ferric-rhodotorulic acid (A. Stintzi and J. M. Ketley, unpublished data) (21), but studies have demonstrated that *C. jejuni* cannot utilize this siderophore for growth (22, 23).

No prior functional studies have been reported for FetABCDEF or their homologs. We predict that these six gene products include a putative membrane protein (encoded by *fetA*), a putative ATP-binding cassette (ABC) transporter (encoded by *fetBCD*), and two putative thioredoxins (encoded by *fetEF*). FetA is predicted to contain eight transmembrane domains, a domain of unknown function (DUF2318), and a YHS domain. The genes *fetB* and *fetC* encode domains consistent with ABC transporter permeases, and *fetD* encodes conserved sequence motifs that are vital for ATP binding and hydrolysis. Thus, *fetBCD* is predicted to encode an ABC transporter for active transport of substrates across the inner membrane. Overall, the *fetMP-fetABCDEF* gene cluster is predicted to encode four distinct inner membrane proteins, which would be unusual for an iron uptake system. The genes *fetE* and *fetF* are predicted to encode single-domain, membrane-associated thioredoxin oxidoreductases. Oxidoreductases can mediate transitions between Cys-Cys disulfide and dithiol groups within proteins, a function dependent on a conserved active site motif (CXXC) (24) that is present in the FetE (CPSC) and FetF (CGVC) protein sequences.

To ascribe the phenotypes observed for Δ*fetABCDEF* (10) to specific genes within the cluster and to provide insight into the essentiality of each functional unit for iron acquisition, this study used a genetic approach to test individual *fet* gene deletion strains for sensitivity to iron availability and to the antibiotic streptomycin. Comparable degrees of growth impairment were observed under low iron upon disruption of *fetM*, *fetP*, *fetA*, *fetB, fetC*, and *fetD*. Based on single and double-mutant analyses, we predict that *fetE* and *fetF* encode gene products that function redundantly under iron limitation. All *fet* deletion strains exhibited an increased sensitivity to streptomycin, implicating iron homeostasis as a determinant of growth modality and resistance during streptomycin exposure. Structural biology and biochemical assays allowed further investigation into the function of FetE as a thiol-disulfide oxidoreductase that may act as an oxidase *in vivo*.

## RESULTS

### Sensitivity to iron availability for *C. jejuni* strains

To investigate the contribution of the *fetMP-fetABCDEF* gene cluster components toward growth under different levels of iron availability, gene deletion (Δ) and complemented (^C^) *C. jejuni* strains were constructed for *fetM*, *fetA*, *fetB*, *fetC*, *fetD*, *fetE*, and *fetF* (Fig. S1). To account for the potential redundancy of the putative thioredoxins FetE and FetF, a double deletion (Δ*fetEF*) and corresponding complemented (*fetEF* ^C^) strain were also constructed. Wild-type *C. jejuni* 81–176 was used as a control, and *C. jejuni* strains corresponding to *fetP* (Δ*fetP* and *fetP* ^C^) and the six gene *fet* cluster (Δ*fetABCDEF* and *fetABCDEF* ^C^) were used as standards in growth curve experiments (10, 11).

**Fig 2:**
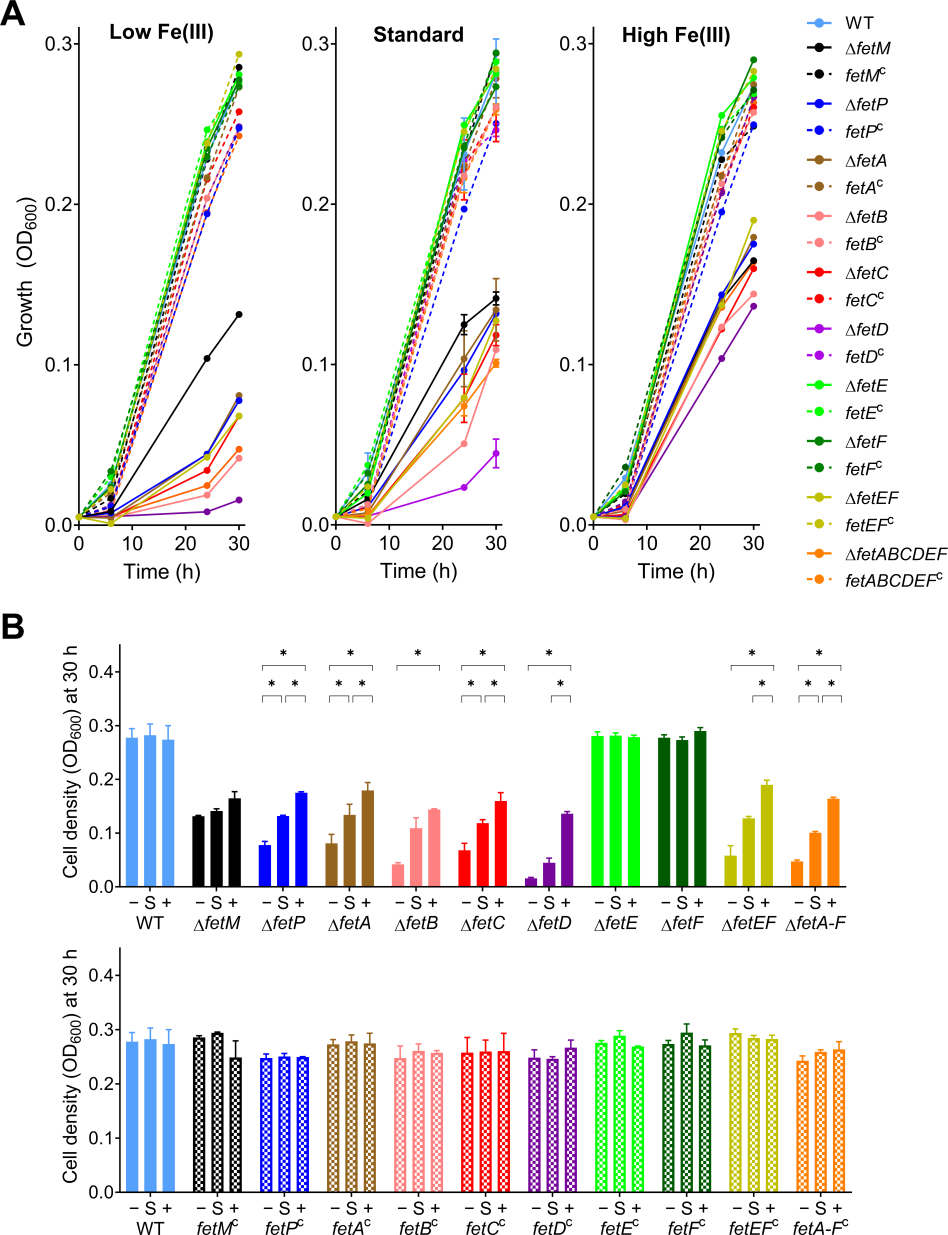
(**A**) Growth by monitoring OD_600_ for *C. jejuni* gene deletion and complemented strains under different levels of iron availability. *C. jejuni* strains were cultured under depleted (−, MH with 5 µM DFO), standard (S, MH), or high (+, MH supplemented with 100 µM FeCl_3_) iron availability. Each strain was assayed in triplicate except for WT, which was cultured for every growth experiment and hence was assayed with 18 biological replicates. Mean values are plotted with error bars representing standard deviation. (**B**) Cell density at the 30-h time point. Growth differences upon changing iron availability were compared for each strain with applied multi-strain comparison correction through two-stage step-up unpaired *t* tests (Benjamini, Krieger, and Yekutieli with 1% desired false discovery rate): *P < 0.001.

**Fig 3:**
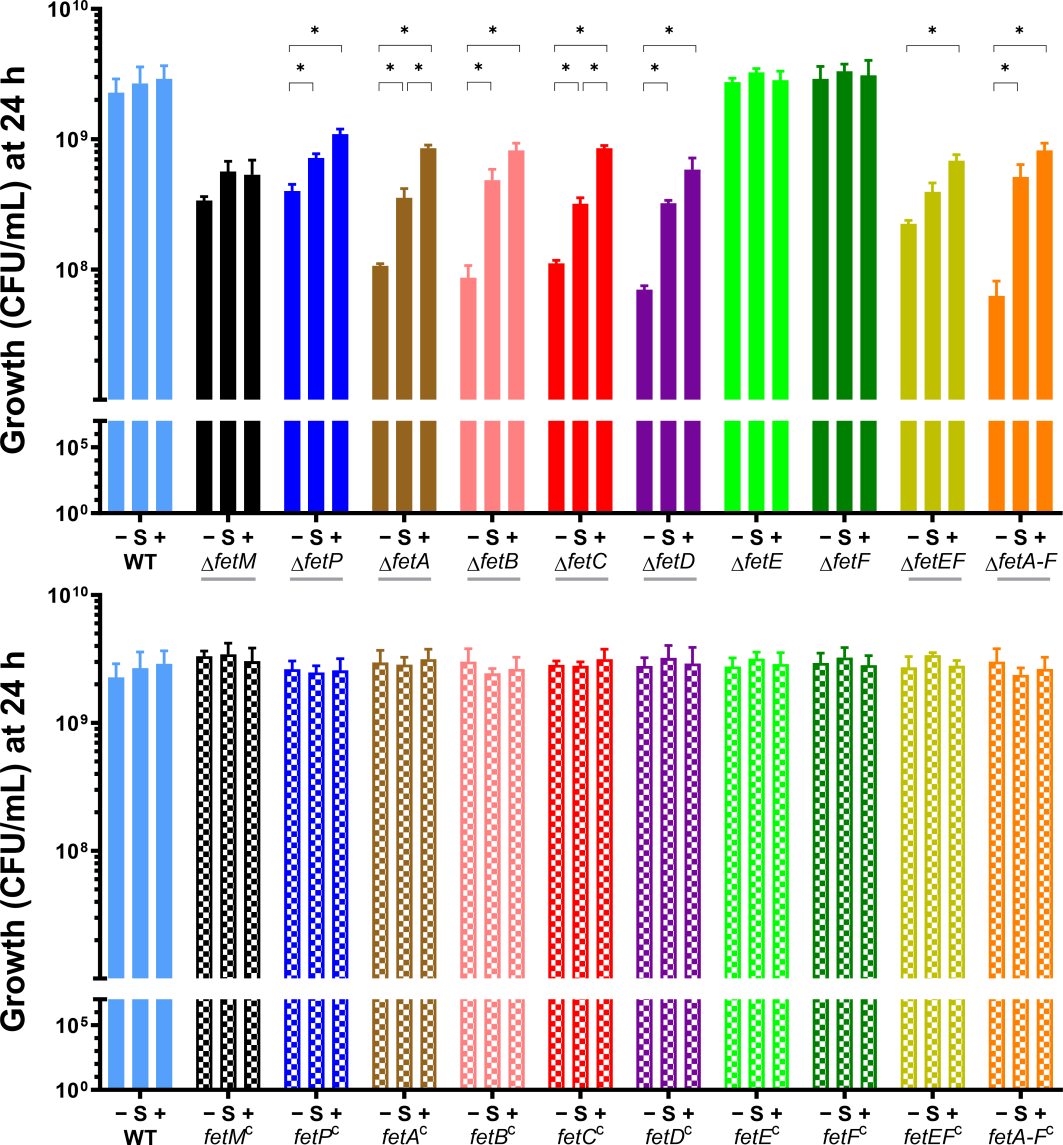
Growth of *C. jejuni* gene deletion and complemented strains over 24 h under different levels of iron availability, as monitored by CFU. *C. jejuni* strains were cultured under depleted (−, MH with 5 µM DFO), standard (S, MH), or high (+, MH supplemented with 100 µM FeCl_3_) iron availability. Each strain was assayed in triplicate except for WT, which was cultured for every growth experiment and hence was assayed with 18 biological replicates. CFU/mL was determined for each culture by dilution plating (five technical replicates). Mean values are plotted with error bars representing standard deviation. Statistical comparison for each deletion mutant versus wildtype was first performed using unpaired *t*-tests: gray underlined strains indicate significance after Bonferroni correction with *P* < 0.005. Growth differences upon changing iron availability were then compared for each strain with applied multi-strain comparison correction through two-stage step-up unpaired *t*-tests (Benjamini, Krieger, and Yekutieli with 1% desired false discovery rate): *P < 0.001.

All *C. jejuni* strains were cultured in iron-restricted Mueller-Hinton (MH) broth, standard MH broth, and iron-supplemented MH broth (Fig. 2 and 3). Iron restriction was achieved by supplementing MH broth with the high-affinity ferric iron chelator desferrioxamine B (DFO), a siderophore that cannot be used by *C. jejuni* as an iron source. Iron supplementation was achieved by supplementing MH broth with 100 µM ferric chloride. As the iron content in MH medium varies between brands and product batches (25), all growth experiments used a single batch of MH medium, and the DFO concentration was optimized to 5 µM from a test range of 0–20 µM (data not shown). The total Fe content of the standard growth medium was measured at 7 µM by inductively coupled plasma mass spectrometry (ICP-MS). The growth of *C. jejuni* strains under different levels of iron availability was monitored by OD_600_ (Fig. 2A) and colony forming units (CFU, Fig. 3). The sensitivity of individual strains to changes in iron availability was assessed by comparing cell densities (OD_600_) after 30 h of growth under each condition (Fig. 2B).

Growth defects demonstrated by gene deletion mutants were fully restored to that of wildtype by ectopic chromosomal complementation. Overall, the trends in growth observed by OD_600_ (Fig. 2) were consistent with those observed by CFU (Fig. 3), with complementation diminishing the possibility that these phenotypes resulted from polar effects on downstream genes. The phenotypic trends under iron limitation observed for the *C. jejuni* strains that were being used as experimental standards (Δ*fetABCDEF*, *fetABCDEF* ^C^, Δ*fetP*, and *fetP* ^C^) were also consistent with those in the original studies (10, 11).

Gene deletion strains Δ*fetM*, Δ*fetP*, Δ*fetA*, Δ*fetB*, Δ*fetC*, Δ*fetD*, and Δ*fetABCDEF* exhibited significant growth defects compared to wildtype by OD_600_ at 24 and 30 h under all levels of iron availability. Additionally, growth after 30 h for Δ*fetP*, Δ*fetA*, Δ*fetB*, Δ*fetC*, Δ*fetD*, and Δ*fetABCDEF* strongly correlated with the level of iron availability for each strain. The growth of Δ*fetM* varied little upon iron restriction or supplementation, indicating insensitivity to iron availability (Fig. 2B). These trends for growth defects and sensitivity to iron availability for Δ*fetM*, Δ*fetP*, Δ*fetA*, Δ*fetB*, Δ*fetC*, Δ*fetD*, and Δ*fetABCDEF* were consistent with CFU data (Fig. 3).

By both OD_600_ and CFU, individual Δ*fetE* and Δ*fetF* strains did not demonstrate growth defects and were similarly insensitive to iron availability when compared to wildtype. The double deletion mutant Δ*fetEF*, however, had significantly reduced growth by OD_600_ and CFU/mL compared to wildtype, Δ*fetE*, and Δ*fetF* under all levels of iron availability and exhibited sensitivity to iron levels.

### Streptomycin sensitivity of *C. jejuni* strains

Deletion of the *fetABCDEF* gene cluster was shown to disrupt streptomycin resistance of *C. jejuni*, which was restored with iron supplementation (10). To investigate the role of each gene in biphasic streptomycin resistance, all deletion strains were assayed for minimum inhibitory concentration (MIC) of streptomycin (Fig. S2). Control wild-type *C. jejuni* cultures exhibited streptomycin-sensitive growth from 0 to 1 µg/mL of streptomycin and streptomycin-tolerant growth from 1 to 4 µg/mL of streptomycin.

All *C. jejuni* gene deletion strains demonstrated increased sensitivity to streptomycin. Δ*fetM*, Δ*fetA*, Δ*fetB*, Δ*fetC*, Δ*fetD,* Δ*fetABCDEF,* and Δ*fetP* exhibited unimodal growth and hence a loss of biphasic phenotype (Fig. S2A). At 1 µg/mL of streptomycin, Δ*fetM*, Δ*fetA*, and Δ*fetD* demonstrated minimal growth similar to Δ*fetABCDEF*, Δ*fetB* and Δ*fetP* demonstrated an intermediate phenotype, and Δ*fetC* was similar to wildtype. Increasing the concentration to 2 and 4 µg/mL of streptomycin, Δ*fetM*, Δ*fetP*, Δ*fetA*, Δ*fetB*, Δ*fetC*, and Δ*fetD* all showed similar growth to Δ*fetABCDEF* (Fig. S2A), whereas Δ*fetE*, Δ*fetF*, and Δ*fetEF* demonstrated intermediate growth, between that of wildtype and Δ*fetABCDEF* (Fig. S2B). At 8 and 16 µg/mL of streptomycin, all strains showed minimal growth. Wild-type biphasic phenotypes and MICs were restored in all complemented strains (Fig. S2C and D).

### Expression of *C. jejuni* FetA protein is independent of FetBCDEF

Due to the strong iron-dependent growth defect observed upon *fetA* deletion and the high level of *fetA* conservation in homologs of the *fet* gene cluster, *fetA* was selected as a gene to characterize further. To examine the protein expression levels of FetA under standard vs iron-limited conditions, a 2×Flag-tagged version of FetA was expressed in the Δ*fetA* and Δ*fetABCDEF* deletion strains using the pRRC-based *fetA* complementation vector (pRRC_1651; Fig. S1K) under the control of the chloramphenicol resistance cassette promoter. FetA^2×Flag^ was able to restore the growth of Δ*fetA*, indicating that the tag had not disrupted function (Fig. S3A). FetA^2×Flag^ was unable to restore the growth of Δ*fetABCDEF*, which expectedly mimicked the growth defect phenotypes of the individual *fetB* to *fetD* deletion and *fetEF* double deletion strains. These strains were then analyzed by western blot, probing for FetA with a monoclonal anti-Flag tag antibody (Coomassie-stained SDS-PAGE loading control: Fig. S3B; western blot: Fig. S3C). No FetA band was observed in the Δ*fetA* and Δ*fetABCDEF* controls. A band was observed for FetA in all tag-complemented strains, with higher protein levels under iron limitation. Full-length FetA is predicted to be ~54 kDa but ran slightly smaller than expected by SDS-PAGE and produced a smeared band when visualized by western blotting, likely due to the large transmembrane region of this protein.

### *C. jejuni* FetE has capacity as a disulfide reductase

In light of our discovery that *fetE* and *fetF* function redundantly under iron limitation, along with the prediction that these genes encode thioredoxins, FetE was selected for further characterization by functional assays and structural analysis. *C. jejuni* FetE was recombinantly expressed in *E. coli* BL21(DE3) and purified. Far-western blot analysis (12) was used to screen for interactions between FetE, FetM, and FetP. While the expected interactions between FetM and FetP were observed, consistent with our previous work (12), no interactions of either FetM or FetP with FetE were detected (data not shown).

**Fig 4:**
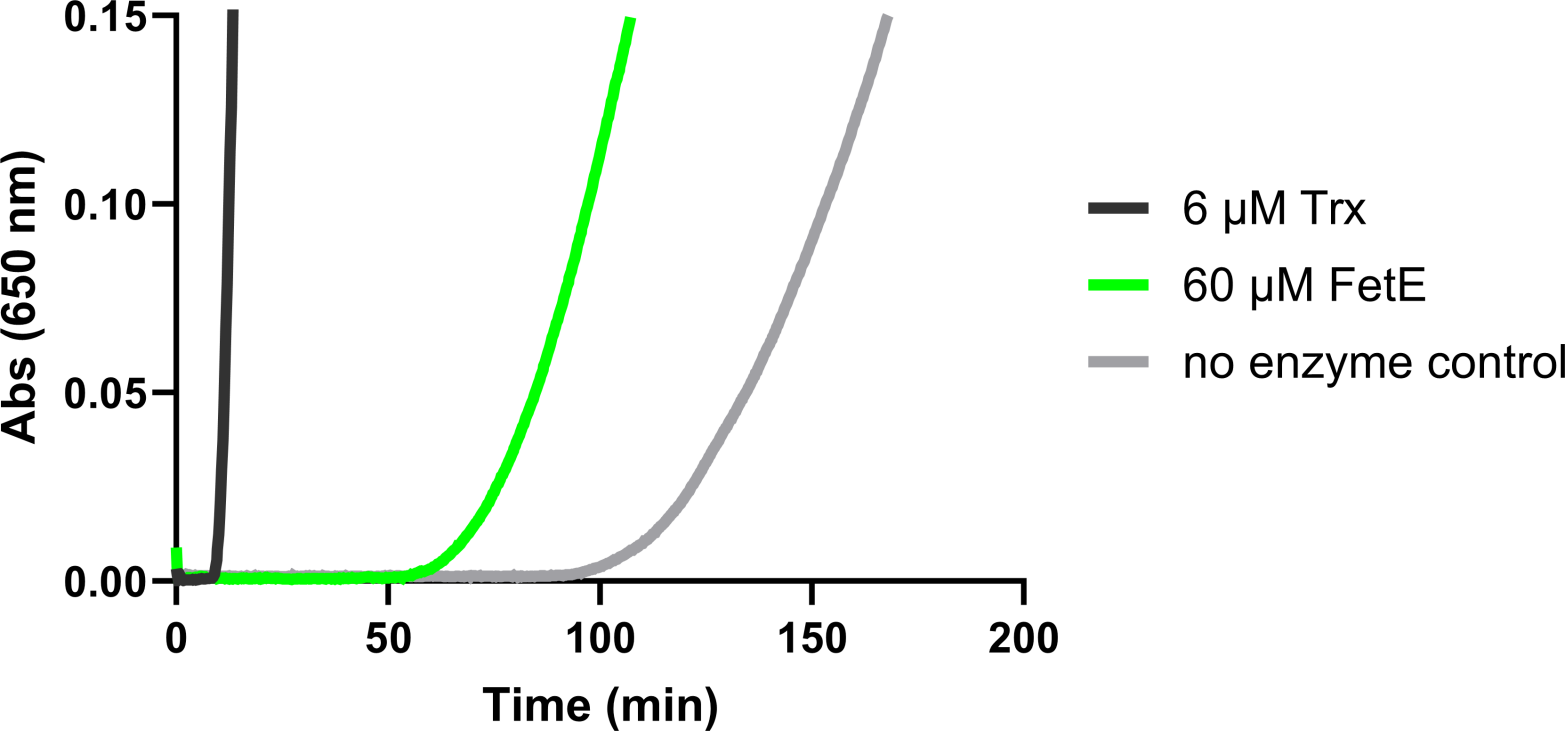
*C. jejuni* FetE exhibits disulfide reductase activity. Reduction of an intermolecular disulfide bond in bovine insulin (0.13 mM prepared in 0.1 M potassium phosphate pH 7.0, 2 mM EDTA, and 0.33 mM DTT) was monitored by an increase in absorbance at 650 nm.

To verify whether *C. jejuni* FetE was capable of reducing disulfide bonds, an insulin disulfide reduction assay was selected as a standard method of thioredoxin characterization (26). The alpha and beta chains of insulin are linked by two disulfide bonds that can be reduced to precipitate the free beta chain. This produces an increase in absorbance at 650 nm that correlates to the rate of disulfide reduction, where baseline insulin reduction by dithiothreitol (DTT) can be increased by the addition of proteins with disulfide reductase activity. This assay was performed for *C. jejuni* FetE with comparison to a standard thioredoxin, *E. coli* Trx, and a blank sample (no protein added) representing baseline insulin reduction (Fig. 4).

The addition of *E. coli* Trx (6 µM) drastically increased the rate of insulin reduction well above the disulfide reductase activity of baseline (no protein), with the rate observed here for *E. coli* Trx being similar to those observed in previous studies (26). The addition of *C. jejuni* FetE (60 µM) also increased disulfide reduction above baseline, albeit to a lesser extent than *E. coli* Trx. Hence, these results indicate a capacity of *C. jejuni* FetE to mediate disulfide reduction.

### *C. jejuni* FetE is structurally related to thioredoxins

The crystal structure of a soluble construct of FetE lacking the lipobox was solved as a monomer to 1.50 Å resolution. FetE consisted of a five-stranded β-sheet with three α-helices on one side and one short α-helix on the other. A structural similarity search of FetE against representative protein folds (PDB25) using the DALI server (27) revealed highest similarity to characterized proteins such as *Streptococcus gordonii* thiol-disulfide oxidoreductase SdbA (the top hit with *Z*-score 17.6; r.m.s.d. 2.1 Å over 133 Cα atoms; PDB ID: 5UM7) and *Mycobacterium tuberculosis* DsbE (*Z*-score 15.5; r.m.s.d. 2.2 Å over 127 Cα atoms; PDB ID: 1LU4). SdbA is an oxidase involved in the formation of disulfide-bonded proteins (28). Similarly, Mtb DsbE functions as an oxidase, which is atypical compared to the reductase role of its Gram-negative DsbE counterparts (29). Other top DALI hits remain uncharacterized. Alignment of 30 unique FetE homolog sequences (*E*-value cutoff = 0.0001) mapped onto the surface of the FetE crystal structure using Consurf (30) revealed conservation of the predicted key catalytic CXXC motif, which are the most highly conserved residues on the surface of FetE (Fig. S4).

### Deletion of *fetEF* affects DTNB reduction by *C. jejuni* cell-free extracts

**Fig 5:**
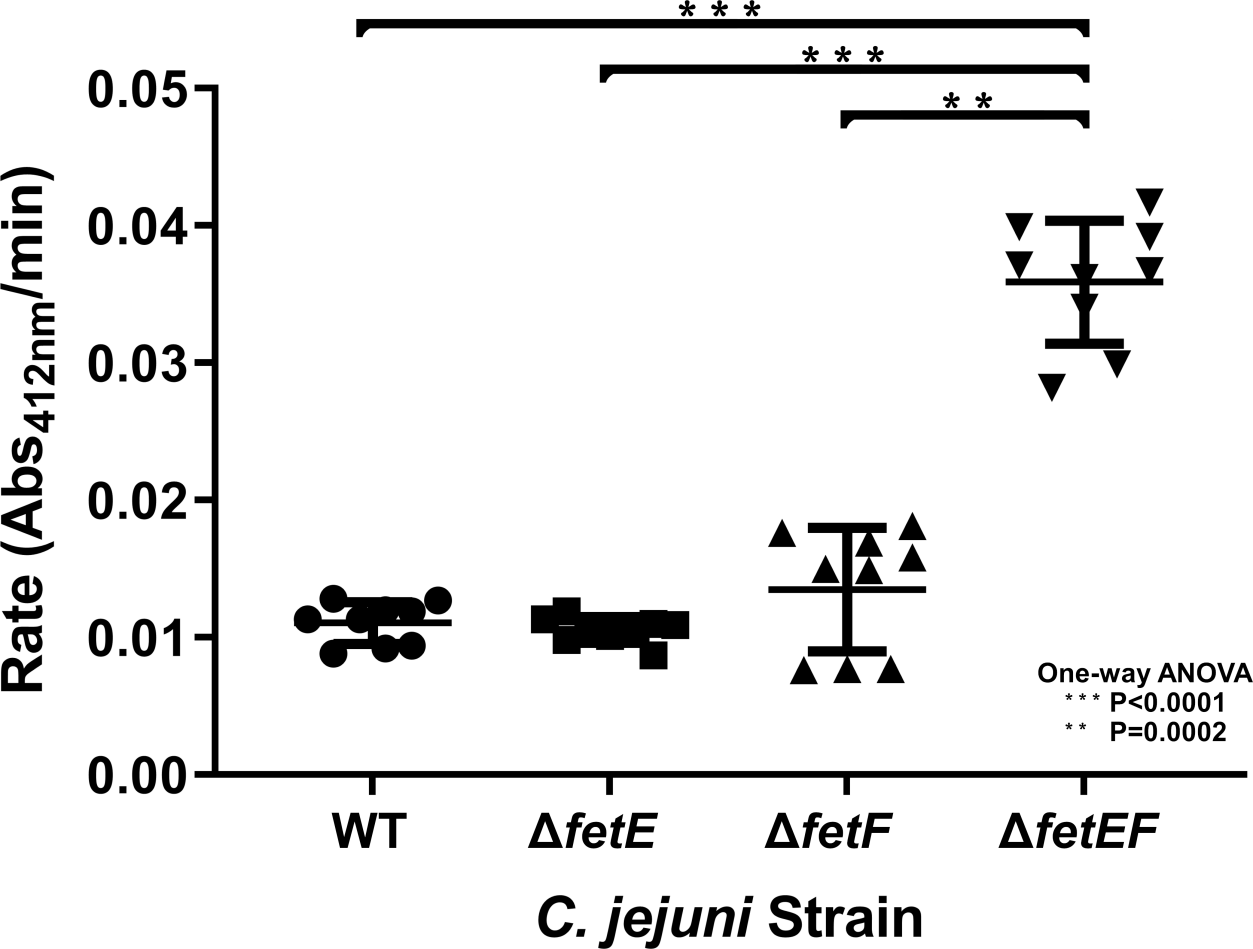
DTNB reduction by *C. jejuni* wildtype, Δ*fetE*, Δ*fetF*, and Δ*fetEF* cell extracts. The overall rate for each strain represents extracts from three mid-log phase cultures incubated for 3 h under iron limitation (5 µM DFO). Reduction of 0.1 mM DTNB in 0.2 mM NADPH and 50 mM Tris-HCl pH 7.2 by each extract was performed three times, with means and error bars representing standard deviation and statistical significance determined by one-way ANOVA.

As predicted thioredoxins, FetE and FetF are hypothesized to mediate disulfide homeostasis in *C. jejuni*. To probe for differences in disulfide reduction capacity, extracts of *C. jejuni* wildtype, Δ*fetE*, Δ*fetF*, and Δ*fetEF* were assayed with the colorimetric agent 5,5′-dithiobis-(2-nitrobenzoic acid) (DTNB). DTNB consists of two aromatic groups linked by a disulfide bond, with disulfide reduction resulting in the production of a thiol anion that absorbs strongly at 412 nm. Cell-free extracts were prepared from *C. jejuni* wildtype, Δ*fetE*, Δ*fetF*, and Δ*fetEF*, normalized for total protein concentration, and then incubated with DTNB and NADH in a cuvette for spectrophotometric determination of the disulfide reduction rate (Fig. 5).

Similar DTNB reduction rates were observed for cell extracts from *C. jejuni* wildtype (0.0110 ± 0.0014 Abs_412_/min), Δ*fetE* (0.0105 ± 0.0009 Abs_412_/min), and Δ*fetF* (0.0135 ± 0.0042 Abs_412_/min) strains. Comparatively, *C. jejuni* Δ*fetEF* cell extract showed a significantly higher rate of DTNB reduction (0.0359 ± 0.0042 Abs_412_/min).

## DISCUSSION

In addressing the extensive global morbidity and socioeconomic burden caused by *C. jejuni*, it is of high priority to gain a deeper understanding of the molecular systems that support virulence during infection, such as the upregulated *fetMP-fetABCDEF* gene cluster. In this study, we systematically deconstructed the *fetMP-fetABCDEF* gene cluster to assess the role of each gene in *C. jejuni* growth during iron scarcity as well as the functionality of one of the predicted oxidoreductase proteins.

Other than the individual thioredoxin deletion strains Δ*fetE* and Δ*fetF*, every tested deletion strain exhibited growth defects. However, no strain was completely devoid of growth, suggesting that other iron uptake systems in *C. jejuni* are responsible for the partial growth observed upon the deletion of *fet* genes. Unexpectedly, the deletion of *fetM* resulted in a growth defect not significantly correlated with iron availability, whereas all other deletion strains with growth defects exhibited greater growth restoration upon increased iron availability. For the Fet cluster, only homologs of FetM have, to date, been shown to directly transport iron (17, 18). Together, this suggests that the other iron-uptake systems are unable to fully compensate for Δ*fetM* growth under ferric chloride supplementation, supporting the key role of FetM in direct iron transport.

The other *fet* genes, with a proposed role in supporting iron transport through FetM, exhibited growth patterns with a stronger dependency on iron availability. Full growth restoration was not observed upon iron supplementation, possibly due to the low solubility (and hence lower bioavailability) of ferric chloride. However, the high sensitivity observed for Δ*fetA*, Δ*fetB,* Δ*fetC,* and Δ*fetD* to iron levels was comparable to that of Δ*fetP*, which directly supports FetM function (12), highlighting an equally important role for the individual FetA and FetBCD proteins under iron limitation. This is reinforced by the high conservation of equivalent genes in all known homologs of the *fetMP-fetABCDEF* cluster across several bacterial phyla (10).

The biochemical function of FetA is not known, but it likely reflects the presence of two predicted periplasmic domains (DUF2318 and YHS). Similar to the thioredoxins FetE and FetF, the DUF2318 domain contains conserved Cys residues. Four of the five Cys residues in DUF2318 constitute two CXXC motifs (CMIC and CISC) that may have a redox role through disulfide formation. The observation of higher FetA protein levels under iron limitation across all 2×Flag-tag-complemented strains suggests that FetA expression may be iron-modulated. The FetA^2×Flag^ construct included the intergenic region between *fetP* and *fetA,* with *fetA* expression under the control of a constitutively expressing Cm promoter. As the Cm promoter is not iron-regulated (31), this suggests the presence of regulatory elements either in the intergenic region between *fetP* and *fetA* present in the complementation construct or within *fetA* itself. Alternatively, *fetA* may be post-transcriptionally regulated. The growth of Δ*fetA^2xFlag-fetA^* was comparable to Δ*fetA^c^* and wild-type strains, demonstrating that tagged FetA is functional. Therefore, the detection of 2×Flag-FetA in both Δ*fetA^2xFlag-fetA^* and Δ*fetABCDEF^2xFlag-fetA^* suggests that FetA is stably expressed in the presence and absence of FetBCDEF.

If FetMP-FetABCDEF represents one iron uptake system in which FetM is the sole iron permease, then the function (and substrate) for the putative ABC transporter encoded by *fetBCD* remains unclear. ABC transporters are a common component of bacterial iron uptake systems, with ATP hydrolysis often driving the passage of a Fe-siderophore complex from the periplasm to the cytoplasm through a channel formed by the two transmembrane proteins (32). Despite homology between *fetB* and *fetC* (28% sequence identity), deletion of either gene resulted in a strong growth defect, indicating that both genes are required for transporter function. This suggests the specific requirement of a FetB-FetC heterodimer for the proper function of this gene cluster and that FetB-FetB or FetC-FetC homodimers are either not formed or cannot sufficiently restore the growth defects of Δ*fetB* or Δ*fetC*.

Individual deletion of *fetE* or *fetF* in *C. jejuni* did not correspond to a growth defect under any level of iron availability, whereas the deletion of both genes (Δ*fetEF*) resulted in a growth defect in the iron-restricted medium. This suggests that *fetE* and *fetF* perform redundant functions to support *C. jejuni* growth during iron restriction, as the presence of either gene is sufficient to maintain growth comparable to wildtype. All *fet* deletion strains exhibited increased sensitivity to streptomycin, suggesting that iron homeostasis is important in antibiotic resistance. However, the streptomycin sensitivity of Δ*fetE,* Δ*fetF*, and Δ*fetEF* were intermediate compared to the other deletion strains. The *fetE* and *fetF* genes are homologs (22% amino acid sequence identity) predicted to encode periplasmic, membrane-associated protein disulfide reductases. We demonstrated that FetE contains a thioredoxin fold and can reduce insulin disulfide. However, the comparatively slow rate of insulin reduction by FetE compared to *E. coli* Trx, the structural similarity to the thiol-disulfide oxidases SdbA and DsbE, and the higher rate of DTNB reduction by extracts of *C. jejuni* Δ*fetEF* than those of wildtype, Δ*fetE*, and Δ*fetF* suggest that the primary role of FetE and FetF may be to act as oxidases *in vivo*. This would be consistent with our previous observation that *C. jejuni* Δ*fetABCDEF* had greater survival than wildtype upon exposure to oxidative stress (10), which suggested that the presence of FetABCDEF increased susceptibility to the deleterious effects of oxidation.

**Fig 6:**
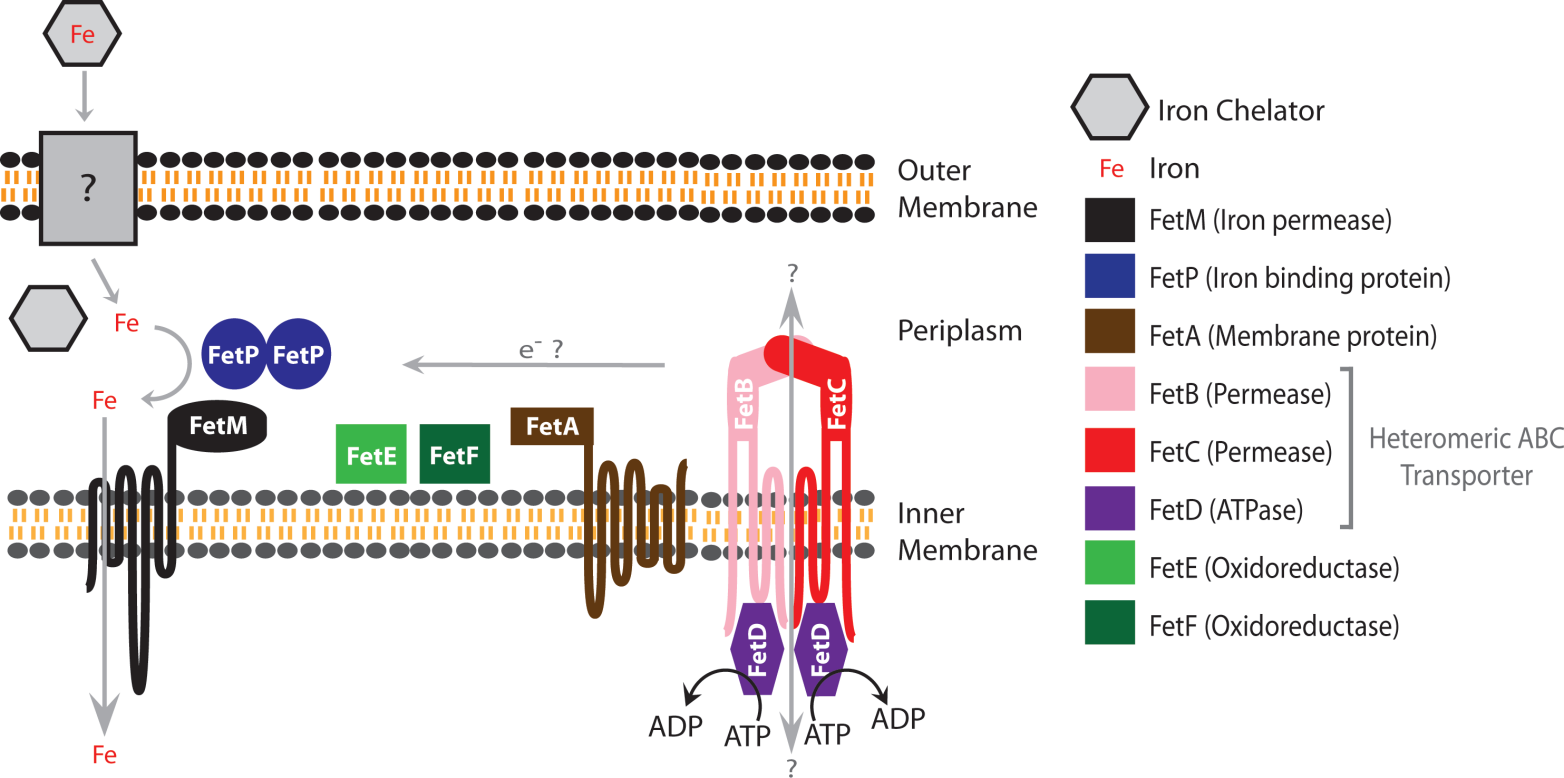
Model of the FetMP-FetABCDEF system based on predicted and known functions in iron transport. Adapted and updated from Liu et al. (10).

Overall, the experimental results described here have advanced understanding on the collective roles of the Fet system components in relation to *C. jejuni* growth under iron limitation. In combining these new findings with an *in silico* investigation and prior literature, we propose an updated model for how the system encoded by *fetMP-fetABCDEF* may function (Fig. 6). In this revised model, an iron-chelator complex first passes through the outer membrane via an as yet unidentified transporter. Upon entering the periplasm, the iron is released from the chelator and transported into the cytoplasm by the cooperative action of the iron-binding protein FetP and the iron permease FetM. Our previous studies in *C. jejuni* and uropathogenic *E. coli* strongly suggest an iron oxidation/reduction-based mechanism for iron transport (11, 12, 18). Based on our studies presented here, we predict that FetE and FetF play overlapping roles in supporting FetMP-based iron transport by actively relaying the necessary reducing power likely sourced from FetB, FetC, and FetD, functioning as a single heteromeric ABC transporter, and through FetA. In the absence of FetM, a major route for iron to cross the inner membrane, growth is impaired irrespective of the presence of other Fet proteins, making this strain more resistant to growth recovery upon iron supplementation. FetA, FetBCD, and FetEF, conversely, each critically support the redox dependency of the FetMP iron uptake function. Hence, the deletion of components from FetABCDEF results in the growth of FetMP-intact strains that are more dependent on overall iron availability. Despite being a double-edged sword, as FetABCDEF increases susceptibility to oxidative stress (10), we demonstrate that this cluster is conserved because it plays an important role in cell growth in conjunction with FetMP.

### Conclusion

The significant global disease burden caused by *C. jejuni* has provided an impetus for research into novel systems important for pathogenesis and pathogenesis-related attributes. The *fetMP-fetABCDEF* genes have stood out as highly upregulated during human infection and in the presence of human fecal extracts, with only recent work bringing the importance of the downstream cluster *fetABCDEF* to light. This study has addressed gaps in knowledge relating to the *C. jejuni fetMP-fetABCDEF* gene cluster through a combined microbial genetics, molecular biology, biochemistry, and structural biology approach. All components of this gene cluster emerged as determinants of growth during iron scarcity, a known virulence-determining factor during *C. jejuni* infection. Expression of the integral membrane protein FetA was shown to be independent of FetBCDEF. FetBCD likely forms a heteromeric ABC transporter essential to the function of the Fet cluster. *C. jejuni* FetE most closely resembles the structure of thiol-disulfide oxidases and demonstrated comparatively poorer disulfide reduction activity. Additionally, cell extracts from the deletion of *fetEF* exhibited the greatest reduction activity, together suggesting that FetE and FetF may function as oxidases *in vivo*.

## MATERIALS AND METHODS

### Design and construction of *C. jejuni* gene deletion and complemented strains

For gene deletion strains, the wild-type gene along with flanking regions was cloned into a pGEM-T plasmid with 45%–90% of the gene replaced with a non-polar *aphA3* kanamycin (Km)-resistance cassette (Table S1). Natural transformation of the modified pGEM suicide vector into *C. jejuni* allowed the replacement of the target gene by homologous recombination at the flanking regions (Fig. S1A through I). For complemented strains, the wild-type gene was cloned into pRRC, which was naturally transformed into its respective *C. jejuni* mutant strain for integration of the gene and an upstream chloramphenicol (Cm)-resistance cassette at one or more of three ectopic loci in the chromosome (33) (Fig. S1J and K).

A list of all strains, plasmids, and primers used during construction for each strain is provided in Table S1. *C. jejuni* 81–176 (clinical isolate from diarrheic patient) was used as the wild-type strain for all experiments (34). Plasmids and strains were verified by PCR analysis followed by Sanger sequencing (Genewiz). The growth conditions for *C. jejuni* and *E. coli*, detailed strain construction protocols, and determination of total iron content in the standard medium by ICP-MS are described in the Supplemental Methods.

### *C. jejuni* growth experiments for sensitivity to iron availability

All *C. jejuni* strains were grown overnight on MH-TV agar plates with Km (deletion strains) or Cm (complemented strains), streaked onto fresh equivalent plates, and then grown for another 6 h. Cells were harvested and resuspended in MH-TV broth (10 mL) to an OD_600_ of 0.0004 (WT), 0.002 (all complemented strains), 0.005 (Δ*fetE*, Δ*fetF*), or 0.02 (all other deletion mutants) to consistently achieve cells in the mid-log-phase (OD_600_ of 0.3–0.6) after a further 18 h of shaking incubation (200 rpm). Mid-log-phase cultures were resuspended in fresh 2× MH-TV and then dispensed into 96 well plates containing equivalent volume aqueous solutions of DFO (10 µM), water, or FeCl_3_ (200 µM) to achieve 200 µL 1× MH-TV starting cultures at an initial OD_600_ of 0.005, corresponding to low iron (MH-TV + 5 µM DFO), standard (MH-TV), and high iron (MH-TV + 100 µM FeCl_3_) conditions. Throughout incubation, growth was monitored by OD_600_ (Thermo Fisher Scientific Varioskan Flash plate reader) at 0, 6, 24, and 30 h, and by CFUs at 0 and 24 h. CFU/mL values calculated for each culture at 24 h were divided by the CFU/mL at 0 h to represent the amount of growth in each culture (CFU_24/0_). All strains were assessed with three biological replicates for each level of iron availability, and CFUs were determined using five technical replicates. Statistical differences were calculated using the Student’s *t*-test.

### *C. jejuni* growth experiments for sensitivity to streptomycin

Full experimental details are described in the Supplemental Methods.

### Expression of 2×Flag-tagged FetA in *C. jejuni*

A C-terminal 2× repeat Flag-tag was inserted into *C. jejuni fetA* by FastCloning (35) using the constitutively expressing complementation vector pRRC_1651 as a template (Fig. S1K) and Q5 DNA polymerase. pRRC_1651 includes 93 C-terminal bp of *fetP,* 82 bp of the intergenic region, and the complete 1,404 bp of the *fetA* gene. *C. jejuni* strains Δ*fetA and* Δ*fetABCDEF* were then complemented with this construct following the method to generate Δ*fetA^c^*, producing Δ*fetA^2xFlag-FetA^* and Δ*fetABCDEF^2xFlag-FetA^*. Successful insertion was confirmed by sequencing purified genomic DNA. Methods to confirm the proper functionality of the tagged FetA variant in the deletion strains are described in the Supplemental Methods.

To examine the expression of tagged FetA in *C. jejuni*, the variant-complemented strains were grown to mid-log-phase in 15 mL MH-TV, resuspended in fresh MH-TV supplemented with or without 10 µM DFO, and pelleted after 3 h of growth. The harvested cell pellets were analyzed by SDS-PAGE and probed by western blot with an anti-Flag antibody (Genscript A01868).

### Expression of *C. jejuni* FetE and other Fet proteins

The expression vector for *C. jejuni* FetE was synthesized by GeneArt (Invitrogen) into pET151/D-TOPO. The DNA sequence encoded an N-terminal 6×His tag, a V5 epitope, a TEV cut site, and residues IDPFT followed by amino acids 22–162 of native FetE (CJJ81176_1655). This vector was transformed into *E. coli* BL21(DE3) for expression and purification, following the protocol for *E. coli* FetA with slight modifications. Cells were induced with 0.25 mM IPTG, lysed in 30 mM Tris, 200 mM NaCl, 5 mM imidazole, and 2 mM TCEP, pH 7.5 and dialyzed into 30 mM Tris, 100 mM NaCl, and 2 mM TCEP, pH 7.5 after His-tag removal with TEV protease. TEV protease was removed with a second nickel resin purification step using dialysis buffer and concentrated. *C. jejuni* FetM and FetP were prepared as part of other studies (11, 12).

### FetE crystallization and structure determination

FetE was crystallized in space group P2_1_ under 1.8 M ammonium sulfate and 0.1 M sodium acetate. Crystals were then soaked in 0.06 M sodium acetate, 1.6 M ammonium sulfate, and 0.5 M sodium iodide, cryoprotected using 30% glycerol, flash frozen with liquid nitrogen, and sent to the SSRL beamline 9-2. A 1.95-Å resolution data set was collected at 1.6 Å wavelength, and iodide phasing was used to successfully solve the crystal structure. This structure was then used for molecular replacement against a non-iodide-soaked 1.5-Å resolution data set collected at CLS beamline CMCF-ID. Data collection and refinement statistics are summarized in Table S2.

### *C. jejuni* cell extracts and DTNB reduction assay

Protocols for the colorimetric DTNB reduction assay with *C. jejuni* cell extracts were adapted from the methods used by Kaakoush et al. (36) to identify *C. jejuni* protein disulfide reductases (36). *C. jejuni* cell extract preparation and the adapted assay are described in the Supplemental Methods.

## Data Availability

The coordinates and observed structure factor amplitudes have been deposited in the PDB under the accession code 8T4C.

## SUPPLEMENTAL MATERIAL

Supplemental material spectrum.03148-23-s0001.pdf Supplemental methods, Fig. S1 to S4, and Tables S1 and S2.

ASM does not own the copyrights to Supplemental Material that may be linked to, or accessed through, an article. The authors have granted ASM a non-exclusive, world-wide license to publish the Supplemental Material files. Please contact the corresponding author directly for reuse.
