# Supplementary material for: Dissecting components of the Campylobacter jejuni fetMP-fetABCDEF gene cluster under iron limitation
Source: Microbiol Spectr. 2023 Dec 14;12(1):e03148-23. doi: 10.1128/spectrum.03148-23 (PMC10783030; doi:10.1128/spectrum.03148-23)
Supplement: Supplemental material — Supplemental methods, Fig. S1 to S4, and Tables S1 and S2. [file spectrum.03148-23-s0001.pdf]

Supplemental Information

Dissecting components of the *Campylobacter jejuni fetMP-fetABCDEF* gene cluster under iron limitation

Tomas Richardson-Sanchez[1†]*, Anson C. K. Chan[1†], Brendil Sabatino[1], Helen Lin[1], Erin C. Gaynor[1§], Michael E. P. Murphy[1§]#

[1]Department of Microbiology & Immunology, The University of British Columbia

Vancouver, BC, Canada

V6T 1Z3

[†]Co-first author – Tomas Richardson-Sanchez and Anson C. K. Chan contributed equally to this work. Author order was determined by greater contribution to experimental design.
[§]Co-final author – Erin C. Gaynor and Michael E. P. Murphy were co-supervisors of this work. These authors contributed equally to project oversight and are listed in alphabetical order.
*Present affiliation: Tomas Richardson-Sanchez, IQVIA, Sydney, Australia

#Address correspondence to Michael E. P. Murphy (michael.murphy@ubc.ca).

Running title: *C. jejuni fet* cluster genes support growth in low iron.

Key words: *Campylobacter jejuni*, iron transport, Fet system genes, thiol-disulfide oxidoreductase, X-ray crystallography.

List of Contents

Supplemental Methods:
1. *C. jejuni* growth conditions
2. *E. coli* growth conditions
3. *C. jejuni* strain construction
4. Determining total iron content of standard medium by ICP-MS
5. *C. jejuni* growth experiments for sensitivity to streptomycin
6. Confirmation of proper functionality of 2xFlag-tagged FetA
7. Insulin reduction assay
8. Preparation of *C. jejuni* cell extracts and DTNB reduction assay

Supplemental Figure S1: Construction of the *C. jejuni* gene deletion and complemented strains.

Supplemental Figure S2: Streptomycin sensitivity for *C. jejuni* gene deletion and complemented strains.

Supplemental Figure S3: The expression of 2xFlag-tagged FetA is iron-regulated and restores the growth of Δ*fetA* but not Δ*fetABCDEF*.

Supplemental Figure S4: The crystal structure of FetE resembles thioredoxins.

Supplemental Table S1: Bacterial strains, plasmids and primers used in this study.

Supplemental Table S2: Data collection and refinement statistics for FetE.

References

Supplemental Methods:

1. *C. jejuni* growth conditions

Unless otherwise indicated, *C. jejuni* was grown at 38 °C under microaerobic and capnophilic conditions (6% O_2, 12% CO_2) produced by a tri-gas incubator (Sanyo) or CampyGen system (Oxoid). The growth medium was MH (Oxoid lot number 2429934) broth or agar plates (1.7% w/v agar) with vancomycin (10 µg/mL) and trimethoprim (5 µg/mL) (MH-TV), and kanamycin (Km, 50 µg/mL) or chloramphenicol (Cm, 20 µg/mL) as necessary.

2. *E. coli* growth conditions

E. coli cultures used in plasmid construction were grown at 37 °C under atmospheric conditions using Luria-Bertani broth or agar plates (1.5% w/v agar). *E. coli* growth media were supplemented with Km (25 µg/mL), Cm (20 µg/mL), ampicillin (Ap, 100 µg/mL), or 5-bromo-4-chloro-3-indolyl-β-D-galactopyranoside (X-gal, 80 µg/mL), where appropriate.

3. *C. jejuni* strain construction

Construction of gene deletion strains Δ*fetM*, Δ*fetA*, Δ*fetB*, Δ*fetC*, Δ*fetD*, Δ*fetE,* and Δ*fetF* was achieved by replacing 45-90% of each gene with the non-polar *aphA3* Km resistance (Km^R) cassette from pUC18-K2 (1). Each gene with approximately 300-400 bp flanking regions was amplified by PCR from *C. jejuni* 81-176 genomic DNA. A polyA tail was added to the PCR product before ligation to a pGEM-T vector (Promega) and transformation into *E. coli* DH5α. Constructs harbouring the resulting plasmid (pGEM_*gene*) were selected using Ap and X-gal plates. pGEM_*gene* was then used as a template for inverse PCR using primers with XbaI and KpnI restriction sites engineered into the 5' ends. The inverse PCR product was digested with XbaI and KpnI and ligated to the *aphA3* Km^R cassette similarly digested out of pUC18-K2 to give pGEM_Δ*gene*. After transformation into *E. coli*

DH5α, constructs containing pGEM_Δ*gene* were selected using Km plates. pGEM_Δ*gene* acts as a suicide vector in *C. jejuni*, incorporating into the chromosome by homologous recombination to replace the target gene *via* the identical 300–400 bp flanking regions. *C. jejuni* 81-176 was naturally transformed with pGEM_Δ*gene* as previously described (2), and Km^R colonies with the gene of interest disrupted by *aphA3* were selected.

Construction of complemented strains *fetM*^{C}, *fetA*^{C}, *fetB*^{C}, *fetC*^{C}, *fetD*^{C}, *fetE*^{C}, *fetF*^{C}, and *fetEF*^{C} was achieved by complementing each deletion mutant with the relevant gene(s) at an ectopic locus in the chromosome. The relevant gene(s) were amplified by PCR from *C. jejuni* 81-176 genomic DNA using primers with XbaI and MfeI restriction sites engineered into the 5' end. In cases where XbaI or MfeI sites could not be used, sites were engineered for the isoschizomers SpeI or EcoRI, respectively. The PCR product was ligated to a similarly digested pRRC vector (3) to give pRRC_*gene*, which was transformed into *E. coli* DH5α. Constructs containing pRRC_*gene* were selected using Cm plates. pRRC is an integration vector that inserts the cloned gene along with an upstream Cm resistance (Cm^R) cassette into one of three 16S ribosomal regions in the *C. jejuni* genome (3). The pRRC_*gene* plasmid was naturally transformed into the corresponding *C. jejuni* gene deletion strain (Δ*gene*). Constructs with both the gene deletion (Km^R) and complementation (Cm^R) were selected on Km and Cm plates. *C. jejuni* strains Δ*fetEF* and *fetEF*^{C} were constructed by these same protocols using the upstream primers for *fetE* with the downstream primers for *fetF*.

4. Determining total iron content of standard medium by ICP-MS

ICP-MS was used to measure the amount of ^{56}Fe in the standard medium (MH-TV) that was used in all *C. jejuni* growth experiments for this study. Three separate batches of standard medium were prepared and each subsampled (500 µL) for use as a replicate. Closed vessel sample digestion was performed in 35% HNO_3 at 110 °C on a hotplate. Solvent was

removed by drying before samples were redissolved in 1% HNO_3 with ^{45}Sc (20 ppb) as an internal standard. ICP-MS was performed using a NexION 300D (Perkin Elmer) equipped with a SC-2 DX autosampler, DXi-FAST micro-peristaltic pump, a cyclonic spray chamber, a triple cone interface, a quadrupole ion deflector and Universal Cell Technology. Calibration was performed using the IV-Stock-4 ICP calibration standard (Inorganic Ventures). All elements were run in reaction mode (using Dynamic Reaction Cell technology) using ammonia as a reaction gas to remove potential polyatomic interferences. The detection limit for ^{56}Fe was determined as 0.356 ppb.

5. *C. jejuni* growth experiments for sensitivity to streptomycin

In assessing streptomycin sensitivity, trimethoprim and vancomycin were not added to MH at any stage. All *C. jejuni* strains were grown to mid-log-phase then resuspended in fresh 2×MH and dispensed into 96 well plates containing equivalent volumes of water or aqueous streptomycin solution at an initial OD_{600} of 0.02. This produced cultures in either unsupplemented medium (MH, positive control) or medium containing doubling concentrations of streptomycin (MH + 0.125–16 μg/mL streptomycin). After 48 h incubation, OD_{600} was determined using a Varioskan Flash plate reader (Thermo Fisher Scientific). Strains were assessed for all streptomycin concentrations with three biological replicates. Growth at each streptomycin concentration was expressed as a percentage of the positive control (unsupplemented MH) and graphed using GraphPad Prism 7.

6. Confirmation of proper functionality of 2xFlag-tagged FetA

Proper functionality of the tagged FetA variant in the deletion strains was determined by comparing growth of the variant-complemented strains to the strains complemented with native FetA and native FetABCDEF and their respective deletion strains, as in main Methods with modifications. Cells harvested from MH-TV plates were resuspended in MH-TV broth

(3 mL) to an OD_{600} of 0.005 (Δ*fetA*, Δ*fetABCDEF* and Δ*fetABCDEF*$^{2xFlag\text{-}fetA}$) or 0.0005 (Δ*fetA*^{c}, Δ*fetABCDEF*^{c} and Δ*fetA*$^{2xFlag\text{-}fetA}$) for overnight starting cultures. Mid-log-phase cultures were resuspended in fresh MH-TV and then dispensed into 96 well plates with 0, 4, 8, 12, or 16 µM DFO to achieve 200 µL starting cultures at an initial OD_{600} of 0.0075. Growth was measured as OD_{600} (Thermo Fisher Scientific Varioskan Flash plate reader) after 24 h of incubation.

7. Insulin reduction assay

The insulin reduction assay protocol was adapted from established methods (4). A solution of potassium phosphate (0.1 M, pH 7.0) was prepared with EDTA (2 mM), insulin solution from bovine pancreas (0.13 mM, Sigma-Aldrich), and DTT (0.33 mM). The insulin reduction reaction was then started by addition of *C. jejuni* FetE (60 µM) or *E. coli* Trx (6 µM, Sigma-Aldrich). To measure baseline insulin reduction, phosphate buffer was added in place of the proteins.

8. Preparation of *C. jejuni* cell extracts and DTNB reduction assay

To prepare *C. jejuni* wild-type, Δ*fetE*, Δ*fetF*, and Δ*fetEF* strains for extract preparation, cultures (25 mL MH-TV) were grown as previously described to achieve robust mid-log-phase growth after 18 h of shaking incubation (200 rpm). At this stage, DFO (5 µM) was added to promote gene expression and cultures incubated for a further 3 h. Cells were pelleted (15000×g, 4 °C, 10 min), supernatant discarded, and each culture resuspended in 10 mL NaCl (150 mM) to wash the cells and increase cell density by a factor of 2.5. This wash procedure (pelleting then resuspension in 10 mL NaCl solution) was repeated three times before cell lysis by six cycles of freezing in liquid nitrogen and thawing. The cell-free extracts were then collected as the supernatant fraction after a final centrifugation step

(15000×g, 4 °C, 10 min) and total protein concentration was determined by Bradford assay. To ensure reproducibility, three biological replicates (cultures) were used for each strain.

Prior to use in the DTNB reduction assay, each cell extract sample was normalized according to total protein concentration and divided into three technical replicates for the assay. 150 mM NaCl was used as the diluent and negative control. For the DTNB reduction assay, DTNB (0.1 mM) and NADPH (0.2 mM) were combined in 50 mM Tris-HCl (pH 7.2) in 1 cm path-length cuvettes. To commence DTNB reduction, normalized *C. jejuni* extracts were added to this mixture and change in absorbance at 412 nm was immediately recorded with a spectrophotometer over 1 min.

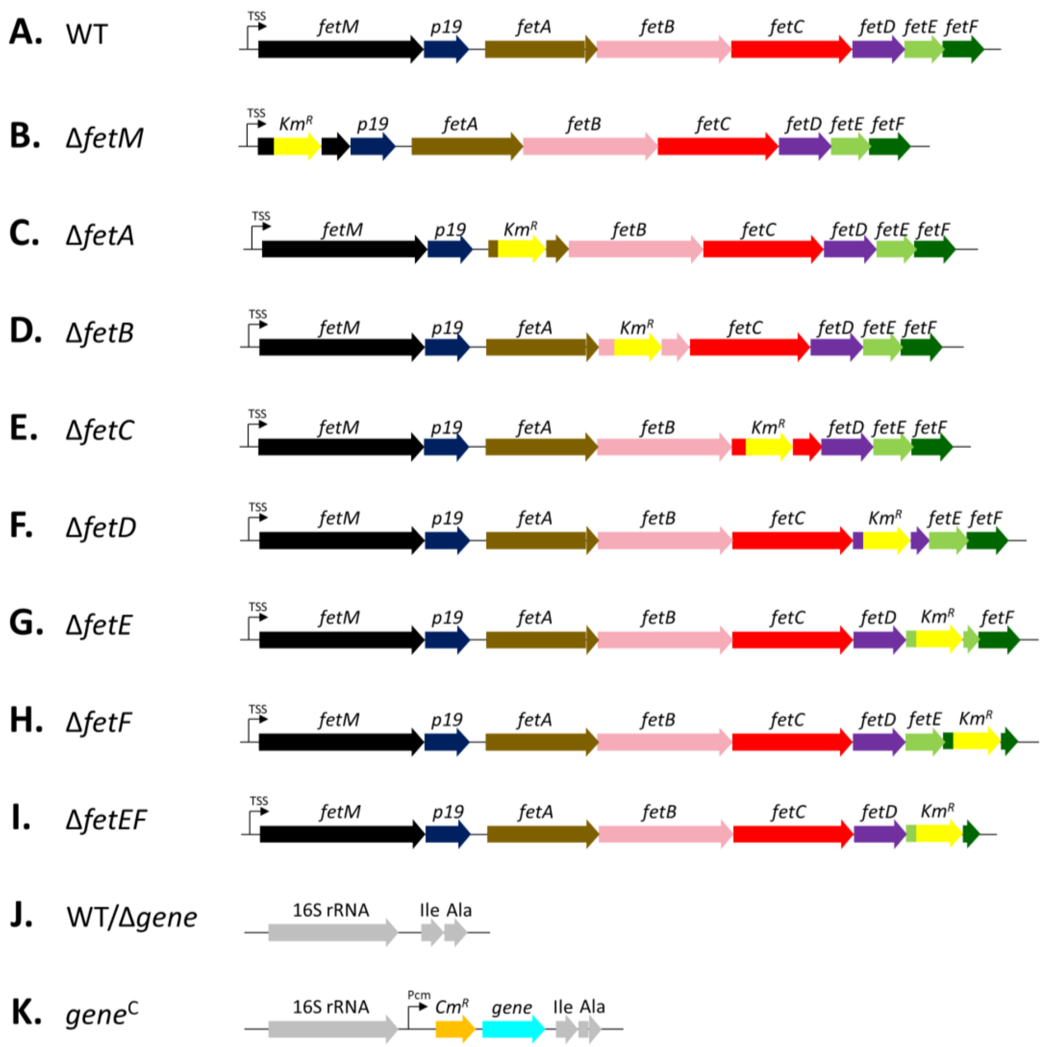

<u>Supplemental Figure S1</u>: Construction of the *C. jejuni* gene deletion and complemented strains. (**A**) *C. jejuni* wild-type (WT) harbours the *1649-1656* gene cluster downstream of a transcription start site (TSS). (**B**-**I**) *C. jejuni* gene deletion mutants were constructed by replacing a portion of the target gene(s) with a kanamycin resistance cassette (Km^R). (**J**) The 16S ribosomal RNA region of *C. jejuni* WT and uncomplemented deletion strains. Directly downstream of the gene encoding for 16S rRNA are genes for $t\text{-}RNA^{Ile}$ (Ile) and $t\text{-}RNA^{Ala}$ (Ala). (**K**) *C. jejuni* complemented strains were constructed by inserting a constitutively-expressing chloramphenicol promoter region (Pcm), chloramphenicol resistance cassette (Cm^R), and the target gene into the 16S ribosomal RNA region of the corresponding deletion strain.

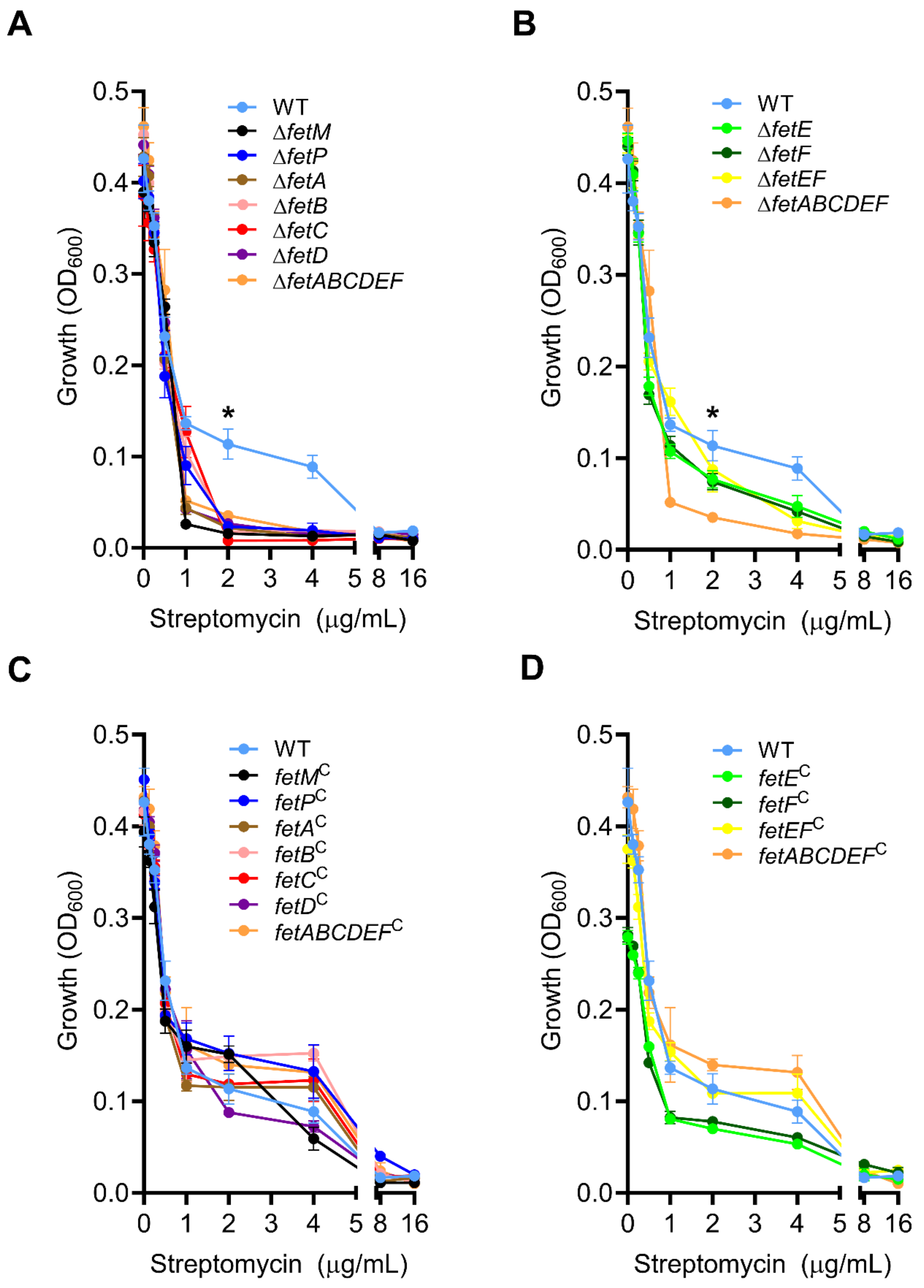

Supplemental Figure S2: Streptomycin sensitivity for *C. jejuni* gene deletion and complementation strains. Deletion strains correspond to (**A**) *fetM*, *fetP*, *fetA*, *fetB*, *fetC*, and *fetD*, and (**B**) *fetE*, *fetF*, and *fetEF*, with comparison to wild-type (WT) and Δ*fetABCDEF*. Complementation strains correspond to (**C**) *fetM*, *fetP*, *fetA*, *fetB*, *fetC*, and *fetD*, and (**D**) *fetE*, *fetF*, and *fetEF*, with comparison to wild-type (WT) and complemented Δ*fetABCDEF*. *C. jejuni* strains were cultured in standard medium (MH) with doubling concentrations of streptomycin. Growth was measured by OD_{600} at 48 h. Mean with error bars representing

standard deviation from three different cultures. *At 2 µg/mL streptomycin, individual *p*-values<0.01 for all deletion strains in a multiple comparisons analysis against WT and for Δ*fetE*, Δ*fetF* and Δ*fetEF* against all other deletion strains. Multi-comparison correction through two-stage step-up method of Benjamini, Krieger and Yekutieli with 1% Desired False Discovery Rate.

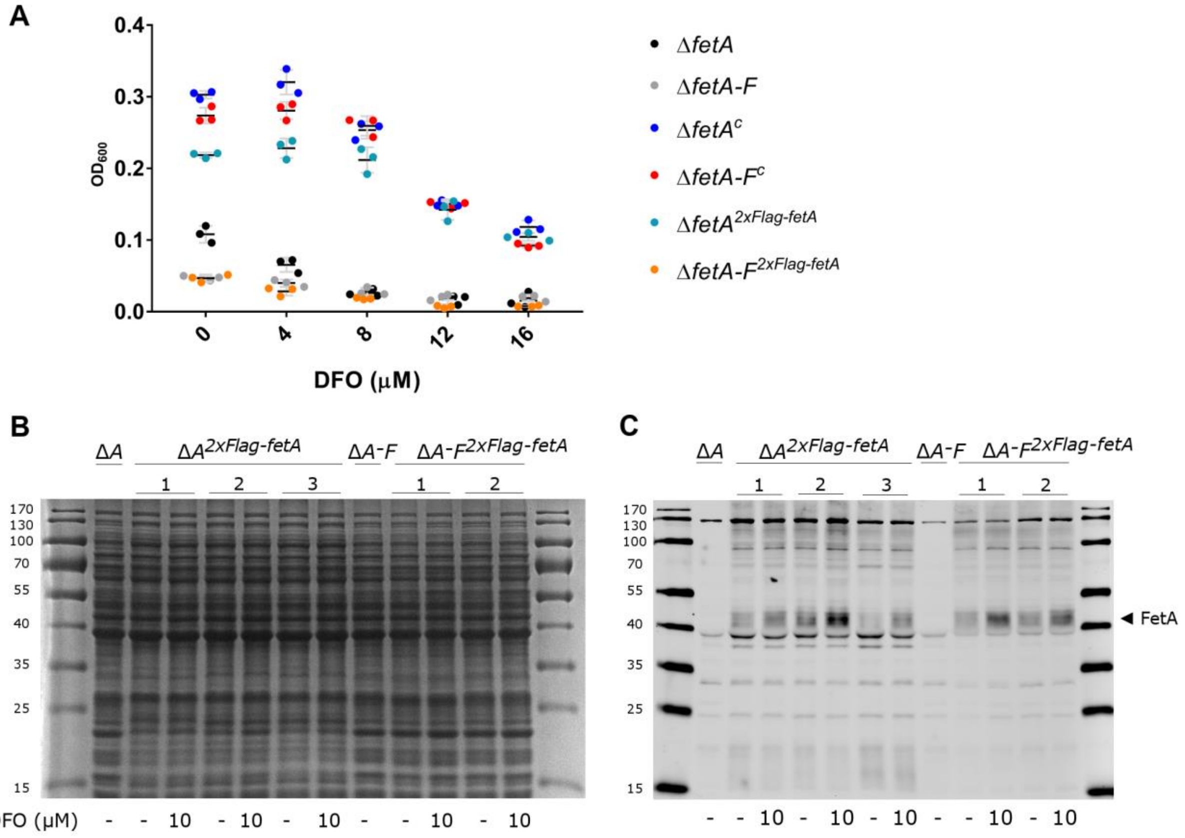

Supplemental Figure S3: (A) The expression of 2xFlag-tagged FetA restores the growth of Δ*fetA* but not Δ*fetABCDEF*. Growth under different levels of iron availability were compared for strains containing a deletion in *fetA* (Δ*fetA*) or *fetABCDEF* (Δ*fetA-F*) and their complemented derivative strains (c = complemented with all deleted genes; 2xFlag-*fetA* = complemented with *fetA* containing a modified two-repeat C-terminal Flag-tag). Iron limitation was achieved by supplementation with increasing DFO. Each strain was assayed in triplicate (dots) with mean (black line) and standard deviation (grey line) shown. (B, C) The expression of FetA is iron-regulated. (B) is an SDS-PAGE gel to show equal sample loading amongst strains. (C) is a western blot using an anti-Flag antibody to demonstrate FetA levels in the deletion strains complemented with the Flag-tagged version of *fetA*. Cells were grown in MH-TV with either no iron limitation or with the addition 10 µM DFO (shown beneath the gels). Biological replicates for the complemented strains are numbered.

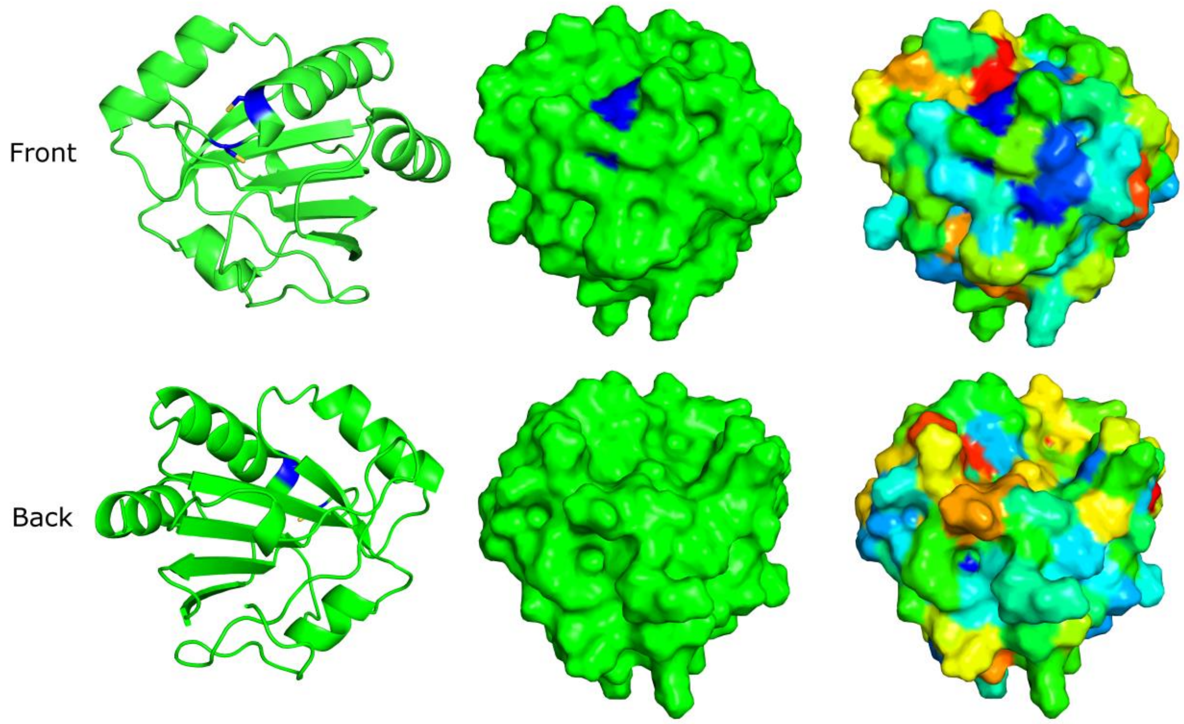

Supplemental Figure S4: The crystal structure of FetE resembles thioredoxins. Cartoon (left) and surface (middle) representations of FetE with CXXC motif highlighted in blue to match conservation. Amino acid conservation analysis mapped to the structure of FetE (right), with most conserved residues in blue, followed by cyan, green, yellow, orange, and then least conserved residues in red.

<u>Supplemental Table S1</u>: Bacterial strains, plasmids, and primers used in this study.

C. jejuni

Strain	Genotype, description, and resistance	Reference or source
81-176	Wild-type isolated from a diarrheic patient	(5)
Δ*fetABCDEF*	81-176 *1651-1656*::*aphA3*; Km^R	(6)
fetABCDEF^C	Δ*1651-56 rrn*::*1651-1656*; Km^R Cm^R	(6)
Δ*p19*	81-176 *1650*::*aphA3*; Cm^R (90% gene removed)	(7)
p19^C	Δ*p19 rrn*::*p19*; Cm^R	(7)
Δ*fetM*	81-176 *1649*::*aphA3*; Km^R (72% gene removed)	This study
fetM^C	Δ*1649 rrn*::*1649*; Km^R Cm^R	This study
Δ*fetA*	81-176 *1651*::*aphA3*; Km^R (87% gene removed)	This study
fetA^C	Δ*1651 rrn*::*1651*; Km^R Cm^R	This study
Δ*fetB*	81-176 *1652*::*aphA3*; Km^R (87% gene removed)	This study
fetB^C	Δ*1652 rrn*::*1652*; Km^R Cm^R	This study
Δ*fetC*	81-176 *1653*::*aphA3*; Km^R (70% gene removed)	This study
fetC^C	Δ*1651 rrn*::*1653*; Km^R Cm^R	This study
Δ*fetD*	81-176 *1654*::*aphA3*; Km^R (74% gene removed)	This study
fetD^C	Δ*1654 rrn*::*1654*; Km^R Cm^R	This study
Δ*fetE*	81-176 *1655*::*aphA3*; Km^R (46% gene removed)	This study
fetE^C	Δ*1655 rrn*::*1655*; Km^R Cm^R	This study
Δ*fetF*	81-176 *1656*::*aphA3*; Km^R (45% gene removed)	This study
fetF^C	Δ*1656 rrn*::*1656*; Km^R Cm^R	This study
Δ*fetEF*	81-176 *1655-1656*::*aphA3*; Km^R	This study
fetEF^C	Δ*1655-56 rrn*::*1655-1656*; Km^R Cm^R	This study
fetA_TM	Δ*1651 rrn*::*fetA_TM*; Km^R Cm^R	This study
fetA_TM_YHS	Δ*1651 rrn*::*fetA_TM_YHS*; Km^R Cm^R	This study
fetA_TM_DUF	Δ*1651 rrn*::*fetA_TM_DUF*; Km^R Cm^R	This study

E. coli

Strain	Genotype and serotype	Reference or source
DH5	F-, φ80d deoR lacZΔM15 endA1 recA1 hsdR17(rK-mK+) supE44 thi-1 gyrA96 relA1 Δ(lacZYA-argF) U169	Invitrogen
BL21(DE3)	F$^–$ ompT gal dcm lon hsdS$_B$(r$_B^–$m$_B^–$) λ(DE3 [lacI lacUV5-T7p07 ind1 sam7 nin5]) [malB$^+$]$_{K-12}$(λ^S)	Novagen

Plasmids

Plasmid	Description and resistance	Reference or source
pGEM-T	PCR cloning vector; Ap^R	Promega
pRRC	*C. jejuni* rRNA spacer integration vector; Cm^R	(3)
pUC18-K2	Source of nonpolar *aphA3* (KanR) cassette; Ap^R Km^R	(1)
pET151_CjFetE	Expression vector for *C. jejuni* FetE	GeneArt (Invitrogen)

Primers

Target gene(s)	Primer ID	Sequence (5' → 3')	Purpose	Amplicon size (bp)
fetM	TRS_**1649**_FOR	GCTTAGAAGAAAACCTTGCGATAC	Initial amplification from WT to construct Δ*fetM*	2490
	TRS_**1649**_REV	GCTAAAGACGCAGCTAGATCA		
	TRS_**1649**_iPCR_KpnI	AATTAA**GGTACC**AGGCTTCATCTTTATACATGCGACG	Inverse PCR of pGEM_1649 to construct Δ*fetM* (KpnI, XbaI)	3992
	TRS_**1649**_iPCR_XbaI	ACAATA**TCTAGA**CAGGAGCCATTCGCATACC		
	TRS_**1649**_compSpeI	TTGATG**ACTAGT**GGCTTAGAAGAAAACCTTGCGATAC	Construction of *fetM*^{C} (SpeI) - used with TRS_1649_REV	2499
	TRS_**1649**_OUTL	GCTATAACCATCTCCTTGAAGAATAC	Verification of Δ*fetM* and *fetM*^{C} by sequencing and PCR	
	TRS_**1649**_OUTR	CCATAGGCATCAAAGTTCCG		
	TRS_**1649c**_middle	CTT CTA CAC CTG CAA ACA ACG		
fetA	TRS_**1651**_FOR	GAACGTGACATTCACGCAC	Initial amplification from WT to construct Δ*fetA*	2287
	TRS_**1651**_REV	CCTTGGACTGCTAAATAAGGATAAAG		
	TRS_**1651**_iPCR_KpnI	CA**GGTACC**CCATTAAAATACTTAGTGGTAAAACACTGATAAG	Inverse PCR of pGEM_1651 to construct Δ*fetA* (KpnI, XbaI)	4087
	TRS_**1651**_iPCR_XbaI	GC**TCTAGA**GCTATAAAGGGATTACTTATTATTTTTCAAATC		
	TRS_**1651**_compXba	CATACT**TCTAGA**GGACGCCATGTTGATGAAG	Construction of *fetA*^{C} (XbaI, MfeI)	1690
	TRS_**1651**_compMfe	TCATAT**CAATTG**GTA GAG AGC AAA AGC GTG G		
	TRS_**1651**_OUTL	GGAAATAGCCGCTGTTTATTTACAAC	Verification of Δ*fetA* and *fetA*^{C} by sequencing and PCR	
	TRS_**1651**_OUTR	GGCATGAAGCAAAATACCTACG		
	TRS_**1651c**_middle	GTCAAGATTTCCCTATTTTTACTAGCTC		
fetB	TRS_**1652**_FOR	CTTGTATGATTTGTGGAGATATGGGC	Initial amplification from WT to construct Δ*fetB*	2112
	TRS_**1652**_REV	CCTAGTTGCTTGGCTAAATTAACTCC		
	TRS_**1652**_iPCR_KpnI	ACT**GGTACC**AGAGACCAAAAGCGTGGC	Inverse PCR of pGEM_*fetB* to construct Δ*fetB* (KpnI, XbaI)	4013
	TRS_**1652**_iPCR_XbaI	ACT**TCTAGA**GCCTTACTAGGTTGCTTGCTTC		
	TRS_**1652**_compXba	ACTCAT**TCTAGA**CTTGTATGATTTGTGGAGATATGGGC	Construction of *fetB*^{C} (XbaI, EcoRI)	2137
	TRS_**1652**_compEcoRI	TCATAT**GAATTC**TCCTAGTTGCTTGGCTAAATTAACTCC		
	TRS_**1652**_OUTL_new	GTG CTG AAG GCA AAG TGA TAA G	Verification of Δ*fetB* and *fetB*^{C} by sequencing and PCR	
	TRS_**1652**_OUTR_new	GGC TAA AAC AAT GCT ATC AAA TTC ATC		
	TRS_**1652c**_middle	GAC ATT TAT TGG CGT AAC AAC ATC AC		

Target gene(s)	Primer ID	Sequence (5' → 3')	Purpose (restriction sites)	Amplicon size (bp)
fetC	TRS_**1653**_FOR	GCGATTTCAAGTCTAATGAGTTCTG	Initial amplification from WT to construct Δ*fetC*	1838
	TRS_**1653**_REV	GTGAGTCTATGCGAAAGTCCTAC		
	TRS_**1653**_iPCR_KpnI	CAT**GGTACC**TTTCATCCCGCTTAGGACTTATC	Inverse PCR of pGEM_*fetC* to construct Δ*fetC* (KpnI, XbaI)	4078
	TRS_**1653**_iPCR_XbaI	CAC**TCTAGA**CTAGTATTGATTTTAGATTTATAGCTGTTTTTATAGCC		
	TRS_**1653**_compXba	CATACT**TCTAGA**CACAGCATTTTCTCAAATCATCAGC	Construction of *fetC*^{C} (XbaI, MfeI)	1411
	TRS_**1653**_compMfe	TTACAT**CAATTG**TGCAAGCCATTCTCCTTCATAGAC		
	TRS_**1653**_OUTL	GCT CTA AAT GCT ATA AGT GAT GCT C	Verification of Δ*fetC* and *fetC*^{C} by sequencing and PCR	
	TRS_**1653**_OUTR	GGT TCA TCT GCG AGT AAA AGC		
	TRS_**1653c**_middle	CAAGCGGAGTAGTTTTAGGCAC		
fetD	TRS_**1654**_FOR	CACTGCGTTTAGCCTTGG	Initial amplification from WT to construct Δ*fetD*	1308
	TRS_**1654**_REV	CACAGCGTAACGATCATTGG		
	TRS_**1654**_iPCR_KpnI	TAC**GGTACC**AAGCCATTCTCCTTCATAGACG	Inverse PCR of pGEM_1654 to construct Δ*fetD* (KpnI, XbaI)	3844
	TRS_**1654**_iPCR_XbaI	TCA**TCTAGA**CA ATC CTG ATT TAG CCA AAT TCG C		
	TRS_**1654**_compXba	ACTCAT**TCTAGA**CACTGCGTTTAGCCTTGG	Construction of *fetD*^{C} (XbaI, MfeI)	1332
	TRS_**1654**_compMfe	TCATAT**CAATTG**CACAGCGTAACGATCATTGG		
	TRS_**1654**_OUTL	GTAGTATTGATCATAGTCACTACTAGCG	Verification of Δ*fetD* and *fetD*^{C} by sequencing and PCR	
	TRS_**1654**_OUTR	GGCAAAATTATAGGTTTTATCACTATTTAAAGC		
	TRS_**1654c**_middle	GTC CAT CAG GAA GTG GAA AAT CAA C		
fetE	TRS_**1655**_FOR	GTA GGA CTT TCG CAT AGA CTC AC	Initial amplification from WT to construct Δ*fetE*	1101
	TRS_**1655**_REV	GCACTTGAAAGGTATTTAGCTGC		
	TRS_**1655**_iPCR_KpnI	CAT**GGTACC**GCAAGTTTTACAGCTTTTCCTAAGG	Inverse PCR of pGEM_1655 to construct Δ*fetE* (KpnI, XbaI)	3898
	TRS_**1655**_iPCR_XbaI	CAT**TCTAGA**GATCGTTACGCTGTGTTTGC		
	TRS_**1655**_compXba	CATACT**TCTAGA**GTAGGACTTTCGCATAGACTCAC	Construction of *fetE*^{C} (XbaI, MfeI)	1126
	TRS_**1655**_compMfe	TCATAT**CAATTG**TGCACTTGAAAGGTATTTAGCTGC		
	TRS_**1655**_OUTL	GACTTGTCTTTCAACAATTTCATCTTATCC	Verification of Δ*fetE* and *fetE*^{C} by sequencing and PCR	
	TRS_**1655**_OUTR	CAAGTATGCTATAAGGAGTTAAGCCTATG		
	TRS_**1655c**_middle	CTTAAGCATTTTGTTCTTGCTCAATG		

Target gene(s)	Primer ID	Sequence (5' → 3')	Purpose	Amplicon size (bp)
fetF	TRS_**1656**_FOR	GGT TGC CCT TCT TGC TTA AAA G	Initial amplification from WT to construct Δ*fetF*	1135
	TRS_**1656**_REV	CTT GTG GCT GTT GTG ATC TTT G		
	TRS_**1656**_iPCR_KpnI	CAT**GGTACC**AAAACCATTGTAGGCAAAATTATAGG	Inverse PCR of pGEM_1656 to construct Δ*fetF* (KpnI, XbaI)	3927
	TRS_**1656**_iPCR_XbaI	CTA**TCTAGA**GCTAAATACCTTTCAAGTGCC		
	TRS_**1656**_compXba	CATCAT**TCTAGA**GGTTGCCCTTCTTGCTTAAAAG	Construction of *fetF*^{C} (XbaI, MfeI)	1159
	TRS_**1656**_compMfe	TCATAT**CAATTG**CTTGTGGCTGTTGTGATCTTTGG		
	TRS_**1656**_OUTL	CGAACCCTTATCTTACAACATGG	Verification of Δ*fetF* and *fetF*^{C} by sequencing and PCR	
	TRS_**1656**_OUTR	CTTAGTGGAAATTCACTATTAGAACATACTATG		
	TRS_**1656c**_middle	CCTATAATTTTGCCTACAATGGTTTTG		
fetA	AC_**1651**Flag_FOR	ATGACGACAAGGACTACAAGGACGACGATGACAAGTAAGCCACGCT TTTGCTCTCTACCAATTG	Insertion of a C-terminal 2x repeat Flag-tag into *C. jejuni fetA* by FastCloning	
	AC_**1651**Flag_REV	TAGTCCTTGTCGTCATCGTCCTTGTAGTCGCCAACATCATTTCTCCT TTGGATTAAAAATTGGG		

Bolded: restriction endonuclease recognition site

<u>Supplemental Table S2</u>: Data collection and refinement statistics for FetE.

Data Collection[a]	FetE (iodide)	FetE (native)
Resolution Range (Å)	36.66-1.94 (1.99-1.94)	46.73-1.50 (1.60-1.50)
Space group	$P2_1$	$P2_1$
Cell dimensions (Å)	*a* = 49.22 *b* = 43.38 *c* = 72.20	*a* = 49.25 *b* = 43.38 *c* = 71.86
	β = 108.3	β = 108.4
Wavelength (Å)	1.600	0.979
Unique Reflections	20879	46005
Completeness (%) [Anom]	97.1 (86.0) [94.7 (75.8)]	99.2 (96.9)
Average I/σI	13.7 (4.3)	16.7 (4.1)
Redundancy [Anom]	4.3 [2.2]	4.2
R_{meas}	0.089 (0.286)	0.052 (0.267)
$CC_{1/2}$	0.995 (0.941)	0.999 (0.935)
**Refinement**		
R_{work} / R_{free}		0.156 / 0.183
No. of waters		272
Average *B*-value (Å^2)		28.5
r.m.s.d. bond lengths (Å)		0.009
Ramachandran plot		
Most-favorable (%)		96.8
Allowed (%)		3.2
Outliers (%)		0

[a] Values for the highest resolution shell are shown in parenthesis

REFERENCES (supplemental data)

1. Menard R, Sansonetti PJ, Parsot C. 1993. Nonpolar mutagenesis of the ipa genes defines IpaB, IpaC, and IpaD as effectors of Shigella flexneri entry into epithelial cells. J Bacteriol 175:5899-906.
2. Gaynor EC, Wells DH, MacKichan JK, Falkow S. 2005. The Campylobacter jejuni stringent response controls specific stress survival and virulence-associated phenotypes. Mol Microbiol 56:8-27.
3. Karlyshev AV, Wren BW. 2005. Development and application of an insertional system for gene delivery and expression in Campylobacter jejuni. Appl Environ Microbiol 71:4004-13.
4. Holmgren A. 1979. Thioredoxin catalyzes the reduction of insulin disulfides by dithiothreitol and dihydrolipoamide. J Biol Chem 254:9627-32.
5. Korlath JA, Osterholm MT, Judy LA, Forfang JC, Robinson RA. 1985. A point-source outbreak of campylobacteriosis associated with consumption of raw milk. J Infect Dis 152:592-6.
6. Liu MM, Boinett CJ, Chan ACK, Parkhill J, Murphy MEP, Gaynor EC. 2018. Investigating the Campylobacter jejuni Transcriptional Response to Host Intestinal Extracts Reveals the Involvement of a Widely Conserved Iron Uptake System. mBio 9.
7. Chan AC, Doukov TI, Scofield M, Tom-Yew SA, Ramin AB, Mackichan JK, Gaynor EC, Murphy ME. 2010. Structure and function of P19, a high-affinity iron transporter of the human pathogen Campylobacter jejuni. J Mol Biol 401:590-604.
